# Environmentally Driven Precision Neurology: A Neurogenomic Perspective

**DOI:** 10.3390/biology15030283

**Published:** 2026-02-05

**Authors:** Mia Yang Ang, Nur Azalina Suzianti Feisal, Muhammad Danial Che Ramli, Zaw Myo Hein

**Affiliations:** 1 Department of Biomedical Sciences, Sir Jeffrey Cheah Sunway Medical School, Faculty of Medical and Life Sciences, Sunway University, Bandar Sunway, Petaling Jaya 47500, Selangor, Malaysia; 2 Sunway Microbiome Centre, Faculty of Medical and Life Sciences, Sunway University, Bandar Sunway, Petaling Jaya 47500, Selangor, Malaysia; 3 MSU Centre for Climate Resilience and Strategy (m-CREST), Management and Science University, University Drive, Off Persiaran Olahraga Section 13, Shah Alam 40100, Selangor, Malaysia; 4 Department of Diagnostics and Allied Health Science, Faculty of Health and Life Sciences, Management and Science University, University Drive, Off Persiaran Olahraga Section 13, Shah Alam 40100, Selangor, Malaysia; 5 Department of Basic Medical Sciences, College of Medicine, Ajman University, Ajman P.O. Box 346, United Arab Emirates; 6 Center of Medical and Bio-Allied Health Sciences Research (CMBHSR), Ajman University, Ajman P.O. Box 346, United Arab Emirates

**Keywords:** environmental neurogenomics, precision neurology, gene-environment interaction, neurological disorders, epigenetics

## Abstract

**The** World Health Organization identifies environmental pollution as a primary global health threat, and its role in the onset and progression of neurological diseases is becoming increasingly clear. In the era of precision medicine, understanding the complex interplay between genetic predispositions and environmental factors is particularly important. The global increase in neurological conditions such as Alzheimer’s disease, Parkinson’s disease, and neurodevelopmental disorders highlights the urgent need for precision neurology. Environmental factors like air pollution, pesticides, and prenatal stress can induce epigenetic changes, including DNA methylation and histone modifications, which alter gene expression and shape disease risk. Advances in neurogenomics, bioinformatics, and artificial intelligence are revolutionizing our ability to decipher these mechanisms, presenting new approaches for personalized diagnostics and interventions. However, significant challenges related to data integration, computational complexity, high implementation costs, and ethical considerations remain. Overcoming these barriers is essential to harness the full potential of environmentally informed precision neurology. This review synthesizes current knowledge on the integration of environmental and genomic data to better predict, prevent, and treat neurological diseases, aiming to alleviate their growing global burden and improve patient outcomes.

## Simple Summary

This review explores how the intersection of our environment and our genetic code influences the onset and progression of neurological diseases. As global cases of conditions like Alzheimer’s and Parkinson’s disease continue to rise, this research highlights the necessity of moving toward precision neurology, a personalized approach that accounts for a person’s lifelong environmental exposures or “exposome”. By integrating genomic data with environmental monitoring and advanced technologies, we can better predict individual risks and develop targeted strategies to protect brain health.

## 1. Introduction

Neurological disorders, including Alzheimer’s disease (AD), Parkinson’s disease (PD), and a range of neurodevelopmental conditions, pose a rapidly escalating threat to global health [1]. The crisis of Alzheimer’s disease is substantial, with an estimated 6.9 million Americans aged 65 and older currently living with Alzheimer’s dementia. This number is projected to nearly double to 13.8 million by 2060, without medical breakthroughs [2]. PD, now recognized as the world’s fastest-growing neurological disorder, saw its prevalence more than double between 1990 and 2015 and is projected to affect over 12 million individuals by 2040 [3]. These alarming trends are driven by a confluence of factors, mainly a globally aging population and the pervasive influence of deteriorating environmental conditions [1].

The state of human health is inevitably linked to the quality of the surrounding environment, with estimates attributing ~70–90% of all human diseases to environmental triggers [4]. This forced precision medicine to evolve beyond its initial, near-exclusive genomic focus [5]. The field now embraces a more holistic framework, recognizing the dynamic interactions between a person’s genetic blueprint, epigenome, and microbiome [6]. While previous research identified key genetic contributors to neurological diseases, the specific environmental factors that initiate or accelerate these conditions remain far more elusive [7].

This knowledge gap has fueled the growth of environmental neurogenomics, a specialized field dedicated to decoding how environmental exposures modify an individual’s genetic risk to shape neurological outcomes [7]. The discipline centers on the gene-environment interaction (GxE), where the combined effect of a genetic variant and an environmental exposure exceed the sum of their individual effects. For instance, evidence shows that airborne pollutants can synergize with *APOE* gene variants to hasten cognitive decline [8], while chronic psychological stress can induce lasting epigenetic changes that predispose individuals to psychiatric illness [9]. Understanding these intricate interactions is the foundation of moving beyond one-size-fits-all approaches to neurological care.

While gene-environment interactions encompass a wide range of environmental exposure, this review focuses specifically on neurodegenerative outcomes associated with air pollution. Particular emphasis is placed on the interaction between particulate matter and genetic susceptibility, especially the APOE-ε4 allele, in driving oxidative stress, neuroinflammation, and impairment of the blood–brain barrier. Fine particulate matter (PM_2.5_), a major component of air pollution, comprises a complex mixture of inorganic elements (Fe, Ni, V, Co, Cu, Cr), organic compounds such as polycyclic aromatic hydrocarbons (PAHs), and secondary aerosols like sulfates and nitrates. This extensively reported yet mechanistically complex relationship provides an important framework for understanding how prolonged exposure to airborne pollutants may contribute to the onset and progression of Alzheimer’s and Parkinson’s diseases. By examining the molecular pathways involved, this review moves beyond largely descriptive epidemiological evidence toward a more integrated mechanistic perspective.

Ongoing challenges in early diagnosis and the limited effectiveness of current treatment of these multifactorial disorders underscore the need for innovative approaches. Neurodegenerative diseases also exert substantial economic and societal burdens through rising healthcare expenditure, reduced workforce productivity, and the emotional toll on caregivers and families [10]. Within the context of precision neurology, this review synthesizes current knowledge on GxE by addressing the brain’s heightened vulnerability, molecular responses to environmental stressors, major risk determinants, and emerging technologies shaping the field. Emphasis is placed on the importance of integrating environmental exposure data with genomic information to support the development of more personalized and effective strategies for neurological care.

**Search Strategy and Selection Criteria** This narrative review is based on a comprehensive literature search conducted across PubMed, Web of Science, and Google Scholar for articles published between January 2010 and January 2026. The search utilized a combination of keywords and MeSH terms, including “environmental neurogenomics,” “gene-environment interaction (GxE),” “exposome,” “neurodegeneration,” “epigenetics,” and “precision neurology,” specifically focusing on Alzheimer’s disease (AD), Parkinson’s disease (PD), and neurodevelopmental disorders. Selection criteria prioritized high-impact peer-reviewed original research, systematic reviews, and meta-analyses. The synthesis includes evidence from human cohort studies, in vivo animal models, and in vitro mechanistic studies to ensure a holistic perspective on the molecular pathways linking environmental triggers to neurological outcomes.

## 2. The Brain’s Unique Vulnerability to Environmental Insult

The central nervous system possesses a unique sensitivity to its environment, a trait rooted in its exceptionally long and complex developmental timeline [11]. This formative period spans from fetal life through the critical windows of infancy and adolescence and into early adulthood. While an individual’s genetic code provides the foundational architecture for the brain, environmental inputs are essential for refining its structure and function [12]. This process, known as experience-dependent plasticity, allows life experiences and sensory inputs to actively shape synaptic connections [13].

The brain’s extended developmental trajectory creates numerous windows of vulnerability, which are specific periods during which it is exceptionally susceptible to disruption [14]. During these times, insults such as exposure to toxins, maternal stress, or nutritional deficiencies can have profound and lasting effects on the brain’s structural and functional integrity [15]. The same plasticity that enables learning also renders the brain vulnerable to adverse inputs that can obstruct neurodevelopment, establishing a potential vulnerability that increases the risk for neurological or psychiatric disorders years or even decades later [16].

Neurological diseases rarely stem from the result of a single cause but emerge from a dynamic interplay between genetic susceptibilities and a lifetime of environmental exposures [17]. Given the need for strong, evidence-based recommendations for doctors, public health programs, and individual strategies to mitigate risk and safeguard neurological health across the lifespan, it is important to understand how various environmental factors, such as local pollutants and climate change, specifically affect the brain [18].

## 3. Core Molecular Mechanisms Linking Environment to Neurological Disease

**Figure 1:**
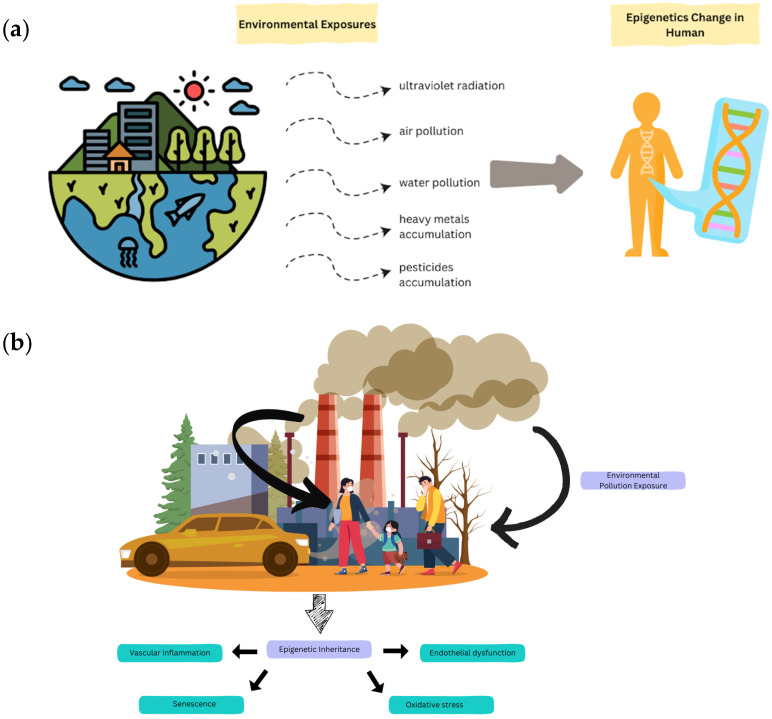
(**a**) Overview of how diverse environmental exposures (e.g., UV radiation, air and water pollution, heavy metals, pesticides) induce epigenetic modifications in human cells, thereby influencing gene expression. (**b**) Illustration of how chronic environmental pollution exposure can lead to epigenetic inheritance, mediating downstream pathological processes including vascular inflammation, endothelial dysfunction, cellular senescence, and oxidative stress.

Air pollution and agricultural pesticides both perturb the neural epigenome, yet they do so through distinct upstream triggers and downstream signatures [19]. Particulate matter (PM_2.5_) drives neurotoxicity through a tractable cascade that links exposure to neurogenomic change and clinical phenotype. For traffic-related and ambient particulate exposure, polycyclic aromatic hydrocarbons and redox-active metal generate reactive oxygen species (ROS) that signal through NF-κB and aryl hydrocarbon receptor (AhR) pathways, producing DNA methylation shifts and histone modification changes in genes governing inflammatory tone and barrier function [20]. Epidemiological and molecular studies have linked PM_2.5_ exposure to hypo/hypermethylation at loci involved in metabolism and stress response such as LEP, PARP1 and to altered chromatin states consistent with sustained inflammatory priming. PM_2.5_ from air pollution is a potent generator of reactive oxygen species. PM_2.5_ refer to airborne particles ≤ 2.5 μm in diameter, typically containing transition metals (Fe, Ni, V, Cr), trace elements, and organic pollutants such as PAHs, which collectively drive oxidative stress and neuroinflammation. pollutant-induced marks can be stable and, under certain conditions, persist across developmental window, echoing broader experimental evidence for transgenerational epigenetic inheritance [21,22]. Figure 1a provides a comprehensive overview of how diverse environmental factors can initiate these crucial epigenetic changes. As depicted in Figure 1b, chronic pollution can lead to epigenetic inheritance that mediates various downstream pathological processes. Collectively, the chain PM_2.5_ to ROS/NF-κB-AhR to methylation or histone changes (LEP, PARP1) to microglial priming and endothelial dysfunction to BBB leakage and neuronal injury to AD-related pathology and cognitive decline that captures the mechanistic bridge from exposure to phenotype.

In comparison, pesticide exposure more commonly initiates mitochondrial impairment and disruption of cholinergic signaling, with epigenetic modifications emerging as secondary consequences of oxidative damage and compromised cellular energetics [23]. Organochlorine compounds have been linked to epigenetic patterns associated with increased susceptibility to Alzheimer’s disease (AD), while agents such as rotenone and paraquat inhibit mitochondrial complex I, elevating reactive oxygen species and inducing DNA methylation and histone modifications that alter dopaminergic survival pathways implicated in Parkinson’s disease (PD) [24]. Collectively, these findings suggest distinct mechanistic signatures, airborne pollutants predominantly drive epigenetic programs related to inflammation and blood–brain barrier integrity, whereas pesticides preferentially shape epigenetic trajectories affecting neuronal metabolism and synaptic resilience. As population-scale epigenome-wide association studies and reference epigenomic resources continue to expand such as epigenome-wide association studies (EWAS) Atlas and Roadmap Epigenomics, cross-exposure comparisons are expected to refine the identification of clinically meaningful, exposure-specific epigenetic profiles [25,26].

An equally important factor is exposure timing. Prenatal and early postanal periods, characterized by dynamic chromatin remodeling and active neurodevelopmental processes, are particularly vulnerable to epigenetic perturbations induced by pollutants and pesticides [27]. Alterations arising during these sensitive windows may establish long-lasting susceptibility patterns that extend into adolescence and adulthood, underscoring the importance of integrating developmental epigenomic data with longitudinal assessments of environmental exposure [28]. For example, tobacco smoke-induced oxidative stress triggers altered histone modifications and decreased histone deacetylase (HDAC) activity, leading to increased expression of proinflammatory cytokines [29]. While numerous studies have demonstrated that pollutants can induce aberrant DNA methylation and histone modifications, establishing a direct causal link between a specific exposure, a precise epigenetic change, and subsequent disease remains challenging due to the subtle and cumulative nature of these alterations.

Beyond epigenetics, a common pathway for neurotoxicity is the induction of oxidative stress and neuroinflammation. The blood–brain barrier (BBB) tight junction proteins (claudins and occluding) and endothelial glycocalyx form a dynamic interface that can be compromised by pollution-derived oxidative and inflammatory signals [30]. Fine particulate matter (PM_2.5_) and its soluble components can access the central nervous system via hematogenous circulation or through olfactory pathway. Once within neural tissues, particle-induced reactive oxygen species (ROS) generation and cytokine signaling activate matrix metalloproteinases, leading to degradation of tight junction proteins and increased paracellular permeability of the BBB. Microglial activation further amplifies barrier disruption, creating a self-reinforcing cycle that facilitates continued entry of toxins and immune cell infiltration.

PM_2.5_ is a strong inducer of oxidative stress, causing damage to neuronal DNA, lipids, and proteins [31]. These molecular insults promote the accumulation of amyloid-beta (Aβ) plaques [32] and tau neurofibrillary tangles [33], which are defining pathological features of Alzheimer’s disease. In parallel, particulate exposure activates resident microglia, stimulating the release of pro-inflammatory cytokines and sustaining chronic neuroinflammation, that accelerates neuronal injury [34]. Disruption of BBB integrity appears to be an early and critical event in this cascade, as the small aerodynamic diameter of PM_2.5_ enables particles to bypass this protective barrier through systemic circulation or olfactory transport [35,36,37]. Compromised barrier function permits both particulate matter and circulating toxicant to enter the brain more readily, thereby intensifying inflammatory signaling and neurotoxic outcomes.

Although exact exposure thresholds differ among experimental models, epidemiological data consistently shows that elevated PM_2.5_ concentrations are associated with increased dementia incidence and greater vascular contributions to cognitive impairment, supporting a link with BBB disruption and subsequent parenchymal damage [38]. Genetic background further modulates these barrier-related effects. The APOE gene, which plays a key role in lipid metabolism, neuronal maintenance, and amyloid-β clearance, has been associated with heightened vulnerability to pollution-related cognitive decline, with individuals carrying the ε4 allele exhibiting stronger relationships between air pollution exposure and neurodegenerative outcomes [39]. At the mechanistic level, APOE-ε4 is thought to intensify lipid oxidative damage, compromise vascular stability, and impair the removal of amyloidogenic peptides, thereby magnifying the consequences of pollutant-induced BBB injury [40].

Oxidative injury and neuroinflammatory signaling function as tightly coupled processes in air pollution-induced neurotoxicity. ROS produced by PM_2.5_ and associated components damage nucleic acids, membrane lipids, and proteins, disrupting mitochondrial integrity and synaptic regulation [41]. This redox imbalance activates NF-κB-mediated transcription, inflammasome pathways, and cytokine release, which prime microglial cells and sustain inflammatory activity within neural tissue. In Alzheimer’s disease, oxidative stress accelerates amyloid-β generation and aggregation as well as tau reinforcing cycles of proteotoxic burden. Comparable mechanisms are observed with tobacco smoke exposure, where histone deacetylase modulation and altered histone marks interact with oxidative pathways to enhance cytokine expression. These relationships exhibit threshold and amplification effects rather than linear progression. Modest increases in oxidative pressure can shift microglia from a regulatory state toward sustained activation, with activated immune cells generating additional ROS that further intensify the initial insult. This feed-forward interaction helps explain how prolonged low-to-moderate exposures translate into meaningful disease risk over time, particularly when compounded by genetic predisposition and compromised BBB integrity [42].

Evidence from human cohort studies indicates that exposure to ambient air pollution is associated with faster cognitive decline, with more pronounced effects observed among individuals carrying the APOE-ε4 allele [43]. Parallel findings have been reported in populations with cardiometabolic comorbidities, where similar gene-environment interaction patterns contribute to elevated dementia risk [44]. Together with converging epidemiological and experimental data, these observations increasingly support a causal relationship between air pollution and Alzheimer’s disease (AD) pathogenesis. At the mechanistic level, APOE-ε4 is thought to impair amyloid-β clearance, increase susceptibility to lipid oxidative stress, BBB permeability, and microglial activation relative to non-ε4 genotypes. This convergence produces a molecular profile marked by oxidative imbalance, elevated inflammatory mediators, degradation of barrier-associated proteins, and epigenetic modification that reinforce sustained inflammatory signaling [45].

From analytical standpoint, combining longitudinal exposure characterization within an exposome framework with integrated multi-omics profiling offers a powerful strategy to clarify causal pathways underlying APOE-pollution interactions [46]. Advances in methylation-based risk indices and biomarker panels reflecting neuroinflammatory activity provide practical endpoints for evaluating preventive or neuroprotective interventions in clinical and population studies. In parallel, artificial intelligence and machine learning approaches that integrate genetic profiles, exposure histories, and clinical phenotypes show promise for predicting individual vulnerability and guiding targeted intervention strategies within genetically defined subgroups. Collectively, these developments position the APOE-PM_2.5_ interaction as a compelling model for precision neurology, combining mechanistic insight with translational feasibility [47].

Taken together, available evidence indicates that prolonged exposure to PM_2.5_ contributes to the progression of neurodegenerative pathology through mechanisms involving oxidative injury, sustained neuroinflammatory signaling, and impairment of BBB integrity, with heightened susceptibility observed among carriers of the APOE-ε4 allele [48]. A notable strength of this body of work is the reproducibility of associations across both population-based studies and experimental systems, reinforcing biological credibility. Nevertheless, much of the epidemiological evidence remains observational and often depends on spatially averaged pollution metrics, which constrains causal interpretation and increases the likelihood of exposure misclassification. Variability in particulate composition, differences in critical exposure windows, and inconsistencies in outcome measurement further contribute to divergent effect estimates across studies [49]. Although several large cohorts demonstrate stronger associations among APOE-ε4 carriers, other investigations report more modest interaction effects, implying that genetic risk may be influenced by concurrent exposures, underlying health status, and social or environmental context [50]. These disparities underscore the importance of developing integrated exposomic and genomic frameworks capable of disentangling pollutant-specific effects and genotype-dependent vulnerability. The inherent susceptibility of the central nervous system shapes how environmental exposures translate into molecular effects, motivating focused analysis of the pathways underlying neurotoxic outcomes.

## 4. Methodological Approaches in Environmental Neurogenomics

Establishing a definitive link between environmental exposures, genetic susceptibility, and neurological disease presents significant methodological challenges. Proving causality requires a multi-pronged approach that combines robust epidemiological observations with controlled experimental validation [51]. Researchers in this field employ a diverse toolkit to navigate the complexities of GxE interactions.

At the forefront are large-scale epidemiological studies, particularly prospective cohort studies and genome-wide association studies (GWAS), which follow large populations over many years [52]. By collecting detailed data on lifestyle, environment, and health outcomes, these studies can identify statistical associations between specific exposures and disease risk [53]. Case–control studies, which compare the past exposures of individuals with and without a disease, provide valuable retrospective [54]. The integration of molecular data into these studies, also known as molecular epidemiology allows researchers to examine how genetic variants, like the *APOE*-ε4 allele, modify the risk associated with an environmental factor [55].

A more holistic approach is captured by the exposome concept, which encompasses the totality of an individual’s environmental exposures from conception onwards [56]. This framework encourages a shift from studying single-agent effects to assessing the complex mixture of chemicals, pollutants, and lifestyle factors that humans experience [57]. Advanced analytical techniques, such as high-resolution mass spectrometry [58] and wearable sensor technologies [59], are making it increasingly feasible to characterize individual exposomes, offering a more complete picture of environmental influence.

However, observational studies can only demonstrate association, not causation [60]. To test causal hypotheses and dissect underlying biological mechanisms, researchers rely on experimental models. In vitro studies using cell cultures and brain organoids allow for high-throughput screening of potentially toxic substances and the precise analysis of their effects on neural cells [61]. In vivo studies using animal models, such as mice genetically engineered to mimic human diseases like AD or PD, are indispensable [62]. Exposing these animals to specific environmental toxins, like air pollution or pesticides, enables researchers to directly observe the pathological consequences and test the efficacy of potential interventions in a controlled setting [63]. By integrating findings from these diverse methodological approaches, the field can build a stronger, evidence-based understanding of how the environment shapes brain health.

Although interest in GxE research continues to expand, important methodological challenges limit the strength of current evidence. Interaction effects are often modest compared with primary genetic associations, meaning that very large cohorts are needed to achieve sufficient statistical power, requirements that many genome-wide studies do not meet. Environmental exposures are also commonly approximated using indirect indicators or areal-level averages, which increases the risk of exposure misclassification and can dilute true interaction signals. Analytical complexity is further heightened by population structure and unequal ancestry representation, particularly when genetic susceptibility and environmental exposure are not evenly distributed across groups. As a result, many reported GxE findings, especially those generated from single datasets. Should be interpreted with caution and regarded as indicative trends rather than definitive causal relationships.

## 5. Major Environmental Exposures and Gene-Environment Interactions

Among the vast array of environmental factors, ambient air pollution has emerged as a critical threat to neurological health [64]. Chronic exposure to pollutants, particularly PM_2.5_, is increasingly associated with cognitive decline, Alzheimer’s dementia, and vascular dementia [65]. This risk is often amplified by an individual’s genetic makeup; studies show a stronger link between air pollution and cognitive decline in carriers of the *APOE*-ε4 allele, a major genetic risk factor for AD [66]. This GxE interaction demonstrates how environmental exposures can unmask or accelerate an underlying genetic predisposition.

**Figure 2:**
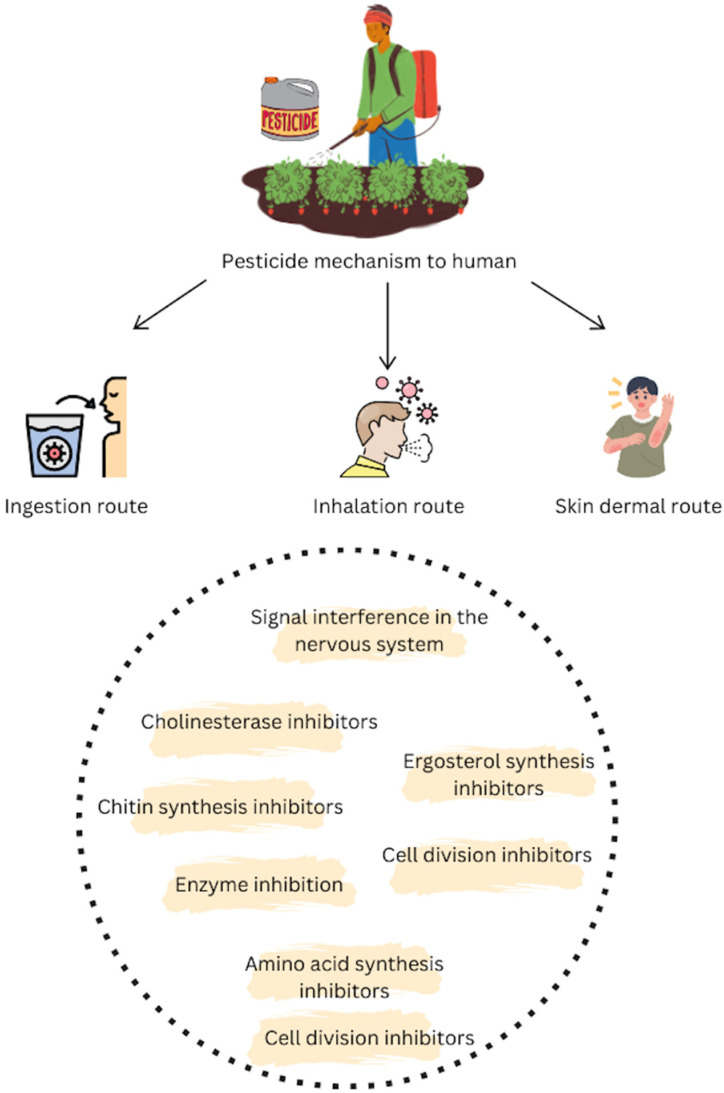
Diagram details routes of pesticide exposure (ingestion, inhalation, dermal contact) and their toxic effects, highlighting neurological mechanisms like signal interference and cholinesterase inhibition, alongside broader cellular targets.

In addition to airborne pollutants, chemicals used in agriculture pose a significant threat, where chronic pesticide exposure has been linked to an elevated risk for both AD and PD [67]. Studies have implicated organochlorine pesticides in AD risk [68], while exposure to agents like rotenone and paraquat is associated with PD, likely by inhibiting mitochondrial function and promoting dopaminergic neuron degeneration [69]. Exposure can occur through ingestion, inhalation, and dermal contact, and these chemicals exert their toxicity through mechanisms such as cholinesterase inhibition, which disrupts nerve signal transmission. Figure 2 illustrates these routes of exposure and mechanisms of toxicity.

The timing of exposure is critical, as the nervous system is acutely vulnerable during prenatal and early-life development [70]. Factors like maternal stress, malnutrition, and toxin exposure can induce lasting epigenetic changes that elevate the risk for neurodevelopmental disorders like autism and schizophrenia [71]. Maternal smoking during pregnancy, for instance, has been shown to cause widespread reduction in DNA methylation in the fetal cortex, disrupting normal cell differentiation [72]. Similarly, early-life exposure to air pollutants has been linked to impaired neurodevelopment and an increased risk for autism spectrum disorder (ASD) [73]. Table 1 summarizes key environmental exposures and their neurological implications.

Decoding the complexity of gene-environment interactions requires a powerful suite of bioinformatics tools and technologies [78]. Foundational to this effort are platforms like STRING [79] and Cytoscape [80], which allow researchers to visualize and analyze protein–protein interaction networks, helping to identify molecular pathways influenced by environmental factors. This network-based approach is supported by large-scale public databases, such as ENCODE [81] and the EWAS Atlas [82], which provide comprehensive annotations of genomic elements and epigenome-wide association data, respectively, forming a critical resource for the research community.

The integration of artificial intelligence (AI) and machine learning (ML) is fundamentally transforming the landscape of environmental neurogenomics [83]. These technologies excel at analyzing vast, high-dimensional datasets to identify patterns and make predictions beyond human capacity. AI-driven models are now being developed to integrate genetic data, environmental exposure profiles, and clinical variables to predict an individual’s susceptibility to neurological disorders [84]. These predictive models can, in turn, power clinical decision-support systems that guide diagnostic and therapeutic choices, paving the way for truly personalized interventions [85].

This technological ecosystem is further enriched by dedicated epigenomic resources. Large-scale projects like the Gene Expression Omnibus (GEO) [86] and the NIH Roadmap Epigenomics Project [87] offer extensive datasets on DNA methylation, histone modifications, and chromatin accessibility. These resources allow researchers to investigate the specific effects of environmental exposures on the epigenetic mechanisms that regulate neural function. Table 2 provides an overview of these key bioinformatics tools and technologies.

Artificial intelligence (AI) and machine learning (ML) methods are increasingly applied to integrate diverse datasets, including genomic and epigenomic profiles, environmental exposure metrics, and clinical phenotypes. These approaches are particularly valuable for identifying non-linear relationships, refining individual risk prediction, and enabling multi-modal inference in complex gene-environment (GxE) systems [88]. In the context of air pollution and neurodegeneration, ML frameworks offer the potential to disentangle how specific particulate matter (PM_2.5_) components such as polycyclic aromatic hydrocarbons, ultrafine particles, and redox-active metals interact with genetic susceptibility to influence Alzheimer’s disease (AD) trajectories [89]. By linking exposure signatures with molecular biomarkers of oxidative stress, neuroinflammation, BBB disruption, and amyloid pathology, computational models may help move beyond association toward mechanistic stratification of risk [90].

Despite this promise, several methodological and translational challenges remain. A major limitation arises from data heterogeneity across platforms and cohorts, including differences in epigenomic technologies such as methylation arrays versus bisulfite sequencing, inconsistencies in air pollution monitoring resolution, and variable chemical speciation of PM_2.5_ across geographic regions [91]. Such variability complicates model transferability and calibration, particularly when attempting to compare exposure-disease relationships across populations.

High-dimensional feature spaces also require careful model regularization and transparent analytical design to minimize overfitting and false discovery, especially when individual exposure effects are modest and pollutants frequently co-occur. For example, separating the neurotoxic contributions of traffic-derived metals from secondary organic aerosols within PM_2.5_ mixtures remains statistically challenging without robust feature selection and biological constraints [92]. Equally important is model interpretability. Clinicians and policymakers must be able to trace predictions back to biologically plausible mechanisms, such as linking elevated metal-rich PM fractions to mitochondrial dysfunction, microglial activation, BBB permeability, and accelerated amyloid-β accumulation. Many current ML architectures remain limited in their ability to provide such mechanistic transparency or causal inference [93].

Of the gene-environment interactions reported to date, the modifying effect of ambient air pollution on outcomes in APOE-ε4 carriers stands out as one of the most reproducible, with support from multiple population studies and experimental models. By comparison, many suggested interactions involving individual candidate genes or particular pollutant categories remain exploratory, frequently derived from small datasets or isolated cohorts. Until these associations are confirmed through independent replication using standardized exposure assessment and supported by mechanistic evidence, they should be interpreted primarily as signals for further investigation rather than established effects.

## 6. Precision Neurology Applications and Future Directions

The ultimate goal of environmental neurogenomics is to translate complex research findings into targeted clinical strategies. In Alzheimer’s disease, this involves integrating environmental risks, such as air pollution and diet, with genetic predispositions like the *APOE*-ε4 allele. Advanced tools like whole-genome sequencing can identify risk-conferring mutations [94], while polygenic risk scores and epigenetic markers are being developed for earlier diagnosis [95]. By combining these data together, clinicians can better predict an individual’s risk and develop personalized interventions, such as tailored nutrition plans [96] and exposure reduction strategies [97] to slow disease progression.

A similar precision framework is being applied to Parkinson’s disease, where interactions between environmental toxins and genetic factors are crucial. Chronic exposure to pesticides such as rotenone and paraquat has been linked to the degeneration of dopaminergic neurons [69]. Precision approaches leverage computational models to analyze multi-omics data, aiming to discover reliable biomarkers like early-stage alpha-synuclein aggregation [98]. This facilitates earlier diagnosis and allows for personalized treatment strategies, including proactive exposure management and neuroprotective interventions.

To move beyond theoretical frameworks, several pilot initiatives are already integrating these data streams. For instance, recent analyses using the UK Biobank cohort have successfully modeled the interaction between APOE-ε4 status and long-term exposure to NO_2_ and PM_2.5_, demonstrating that the genetic risk for cognitive decline is significantly magnified in high-pollution environments compared to genetic risk alone [5]. In Parkinson’s disease, the Genetic Epidemiology of Parkinson’s Disease (GEoPD) consortium has explored how variants in the *CYP2D6* gene—responsible for metabolizing endogenous and exogenous toxins—interact with organophosphate pesticide exposure to drastically increase disease susceptibility. Furthermore, the development of Methylation Risk Scores (MRS) is now being piloted to provide a ‘biological readout’ of cumulative environmental stress, offering a more precise measure of an individual’s ‘neuro-exposomic’ age than chronological age alone [95].

This approach is also highly relevant for neurodevelopmental disorders like autism spectrum disorder (ASD) and attention-deficit/hyperactivity disorder (ADHD) [99]. These conditions are believed to arise from a combination of genetic predisposition and environmental factors during critical developmental windows [100]. Chronic maternal stress, for instance, can elevate cortisol levels, which may cross the placenta and impair fetal brain development [101]. In response, precision neurology aims to mitigate these risks through personalized prevention, such as tailored nutritional support (e.g., folate, omega-3 fatty acids) and stress-management programs for high-risk pregnancies [102].

From a clinical standpoint, the practical application of current gene-environment interaction findings remains constrained. Existing evidence does not yet justify the use of GxE information for personalized clinical decision-making, predictive genetic screening for environmental vulnerability, or tailoring of exposure-specific interventions. Most reported interactions demonstrate relatively small effect sizes, rely on imprecise exposure characterization, and lack fully established mechanistic pathways, limiting their translational readiness. Nonetheless, GxE research holds increasing value for population health and preventive strategies. The identification of genetically sensitive groups such as individuals carrying the [95]-ε4 allele in high-air-pollution environments can guide targeted prevention efforts, inform public health policy, and improve risk communication frameworks. With continued advances in exposome quantification, longitudinal cohort integration, and experimental validation, these interaction models may progressively evolve from tools for population-level risk estimation toward clinically meaningful applications in precision neurology.

## 7. Therapeutic Innovations and Clinical Translation

The insights gained from environmental neurogenomics are not only refining our understanding of disease etiology but are also paving the way for novel therapeutic [103]. A key advantage of this strategy is its potential for drug repurposing, enabling faster translation to clinical use by utilizing compounds with established safety and regulatory profiles. Compared with conventional drug discovery pathways, this approach substantially lowers development costs and shortens timelines, making it especially appealing for tacking neurological conditions influenced by environmental [104]. By elucidating the molecular pathways disrupted by environmental exposures, researchers can identify new targets for pharmacological intervention designed specifically to counteract the harmful effects of environmental insults on genetically susceptible individuals [105]. For instance, if a specific pollutant is found to disrupt a particular histone deacetylase (*HDAC*) in individuals with a certain genetic background, *HDAC* inhibitors could be explored as a targeted therapy for that subgroup [106].

Drug repurposing is another promising strategy accelerated by environmental neurogenomics. By integrating genomic, transcriptomic, and environmental exposure data, computational models can identify existing drugs that may be effective in mitigating the downstream consequences of GxE interactions [91]. For example, drugs with known anti-inflammatory or antioxidant properties could be repurposed to treat neuroinflammation triggered by air pollution exposure, particularly in individuals with a genetic predisposition to an exaggerated inflammatory response [107]. This approach offers a more rapid and cost-effective path to clinical application compared to traditional drug development pipelines.

Despite this promise, the translation of these findings into clinical practice is in its early stages. Clinical trials in environmental neurogenomics face unique challenges. They require robust methods for quantifying lifelong environmental exposures, which can be difficult to reconstruct accurately [108]. Furthermore, the long latency periods of many neurological diseases mean that trials may need to span decades to demonstrate a preventative effect [109]. Nevertheless, innovative trial designs are emerging, such as studies focusing on intermediate biomarkers of disease (e.g., epigenetic modifications, levels of neuroinflammation markers) to provide earlier indications of therapeutic efficacy [110]. The development of more sensitive biomarkers of both exposure and effect is a critical area of ongoing research that will be essential for the successful clinical translation.

Ultimately, the successful translation of environmental neurogenomics into clinical practice requires a structured “precision neurology” pathway. This process would ideally begin with an integrated assessment, where high-resolution geospatial data of a patient’s lifetime pollutant exposure are mapped alongside APOE genotyping and polygenic risk scores. Such data would then feed into an AI-driven decision-support system to categorize individuals into specific risk strata. For a patient identified as high-risk due to the synergy between APOE-ε4 and heavy PM_2.5_ exposure, the clinical output would shift from generic care to “precision prescriptions,” such as the prioritized use of medical-grade HEPA filtration in the home, targeted antioxidant supplementation to bolster glutathione pathways, and early-stage monitoring of neuroinflammatory biomarkers. By moving from broad associations to this type of stratified, evidence-based intervention, the field can transition from theoretical research to a tangible framework for personalized neurological care.

The clinical translation of these findings requires a standardized precision neurology pathway that begins with a multimodal assessment, mapping a patient’s geospatial pollutant exposure alongside *APOE* genotyping and polygenic risk scores to identify high-risk strata via AI-driven decision-support systems. For instance, individuals carrying the APOE-ε4 allele who reside in high-pollution areas could receive “precision prescriptions” such as medical-grade HEPA filtration and targeted antioxidant therapy tailored to their specific gene-environment profile. However, several practical challenges remain, including the high cost of multi-omics data generation, the lack of standardized exposure metrics across different geographic regions, and the computational complexity of integrating high-dimensional environmental datasets into existing clinical workflows. Addressing these barriers through strategic investment and the development of equitable clinical guidelines is essential for moving environmental neurogenomics from a theoretical framework into a tangible tool for individualized patient care.

## 8. Public Health and Policy Implications

The increasing evidence from environmental neurogenomics has significant implications for public health and policy. By demonstrating a causal link between specific environmental exposures and neurological diseases, this research provides a strong scientific basis for regulatory action. A key strength of this evidence is its ability to support stricter air quality standards and pesticide regulations, offering policy makers a clear rationale for intervention. For example, evidence linking PM_2.5_ to an increased risk of dementia, particularly in genetically susceptible individuals, strengthens the case for stricter air quality standards [111]. Similarly, findings on the neurotoxicity of certain pesticides can inform policies regarding their use, restriction, or outright ban to protect public health [112].

From a public health perspective, environmental neurogenomics enables a shift towards a more preventative approach to neurological disorders. By identifying populations at high risk due to a combination of genetic and environmental factors, public health interventions can be targeted more effectively [113]. This could include public awareness campaigns about the risks of certain exposures, promoting lifestyle modifications (e.g., dietary changes to boost neuroprotection), and advocating for urban planning that reduces pollution hotspots [114]. The concept of the neural exposome is also important for encouraging a holistic view of all environmental factors that impact brain health throughout the lifespan and for informing comprehensive public health strategies [115].

Furthermore, this field is crucial for addressing issues of health equity and environmental justice. Previous study showed that marginalized and low-socioeconomic communities are often disproportionately burdened by exposure to environmental neurotoxicants [116]. By providing molecular evidence of the harm caused by these exposures, environmental neurogenomics can be a powerful tool for advocating for policies that protect vulnerable populations and reduce health disparities [117]. The application of this knowledge is crucial to ensure that the benefits of precision neurology do not worsen existing social inequalities. As our understanding of the environmental factors driving neurological disease grows, it becomes a societal imperative to translate this knowledge into policies that protect the brain health of everyone [118].

To effectively translate these findings into societal benefits, several priority policy actions are recommended. These include the implementation of stricter, localized air quality and pesticide regulations specifically within high-risk “pollution hotspots” and the establishment of integrated surveillance programs that combine real-time environmental monitoring with routine neurocognitive screening. Furthermore, there is an urgent need to prioritize funding for longitudinal gene-environment interaction (GxE) cohort studies within marginalized communities to ensure research equity. Finally, developing formal clinical guidelines to integrate environmental risk assessments into standard precision neurology and memory clinic protocols will be essential to transition these insights from public health theory into actionable medical practice.

## 9. Barriers to Implementation

Despite its immense promise, the widespread implementation of environmental neurogenomics faces significant barriers. A primary technical hurdle is the lack of standardization across diverse data types—including genomics, epigenomics, and environmental exposures—which complicates data sharing and integrated analysis [91]. The massive volume and complexity of these datasets also demand advanced computational infrastructure, creating a barrier for many research institutions and healthcare systems, particularly in low-resource settings [119].

Beyond technical issues, economic and policy obstacles persist. The personalized approaches of precision neurology are associated with high costs, stemming from multi-omics data generation and sophisticated computational tools [120]. These expenses make precision neurology inaccessible to much of the global population and strain even well-funded healthcare systems. This is worsened by a lack of cohesive policies and funding frameworks designed to promote the integration of genomic and environmental data into clinical practice and to ensure equitable access [121].

Finally, these challenges are layered with critical ethical, legal, and social considerations. The collection and analysis of highly sensitive genomic and personal exposure data create substantial privacy and security risks [122]. Establishing public trust requires strict ethical guidelines and robust data protection policies. Furthermore, significant questions persist regarding how to equitably translate research findings into practice, lest these innovations unintentionally widen existing health disparities [123]. Advancing the field requires a delicate balance between fostering innovation and upholding ethical responsibility to all members of society. Addressing these scientific, technological, and ethical challenges is essential for realizing the full potential of environmentally driven precision neurology.

## 10. Conclusions

Recent advancements in environmental neurogenomics have clarified how lifelong exposure to environmental stressors interacts with genetic susceptibility to influence the onset and progression of neurological disorders. It drives a necessary shift from a gene-centric view to a holistic appreciation of the lifelong interplay between genetics and environment. The alarming rise in conditions like Alzheimer’s and Parkinson’s is not a matter of genetics alone but is inseparably linked to the physical, chemical, and social worlds we inhabit. By harnessing the power of multi-omics technologies, bioinformatics, and artificial intelligence, precision neurology holds the potential to usher in a new era of personalized medicine, one that offers earlier diagnosis, individualized risk prediction, and targeted strategies for prevention and treatment. However, the path forward is constrained by significant technical, economic, and ethical barriers. Through strategic investment, the development of equitable policies, and dedicated commitment to ethical principles, these challenges can be overcome. By continuing to elucidate the complex connections between our environment and our neurological health, we can transform the future of care, ultimately reducing the immense global burden of these devastating diseases.

To move environmental neurogenomics beyond associative findings toward meaningful translational outcomes, several strategic directions require immediate emphasis. On the research front, priority should be given to building large-scale, longitudinal exposome cohorts that integrate genomic, epigenomic, transcriptomic, and high-resolution environmental data across the lifespan, establishing harmonized and standardized exposure assessment frameworks to strengthen comparability across studies and enhance causal interpretation and discovering and validating reliable molecular and epigenetic indicators that reflect cumulative neurotoxic load rather than isolated exposure events. Concurrently, important clinical and public health efforts should focus on incorporating gene-environment interaction parameters into predictive models for neurodegenerative risk, developing targeted prevention strategies informed by exposure profiles for susceptible populations, and translating multi-omics discoveries into clinically actionable decision-support systems that facilitate early detection and individualized intervention. Collectively, advancing these priorities will be critical for positioning environmental neurogenomics as a practical platform for precision neurology and the promotion of equitable brain health.

## Figures and Tables

**Table 1:** Selected Environmental Exposutes, Associated Neurological Outcomes, and Proposed Mechanisms.

Exposure Type	Exposure Components	Related Diseases	Modification Levels/Gene Targets	Mechanism of Action	References
Air Pollution	NO_2_, PM_2.5_ and PM_10_	Cognitive Decline	*APOE*-ε4 status	Induce inflammation and oxidative stress, exacerbated by *APOE*-ε4.	[43]
	PM_2.5_, PM_2.5–10_, PM_10_, NO_2_ and NO_x_	AD, Vascular Dementia		Contributes to neuroinflammation and oxidative stress, accelerating AD/VD progression.	[74]
	Tobacco smoke	Respiratory Inflammation	Histone methylation	Induces epigenetic changes that alter expression of immune and inflammation genes.	[75]
	PM_10_	Neurological Disorders	Hypomethylation of *LEP*, *PARP1*	Alters expression of genes impacting neural function, DNA repair, and stress response.	[76]
	PM_2.5_	Neurodegeneration	Aβ accumulation, Tau pathology	Promotes neuroinflammation and oxidative stress, accelerating hallmark AD pathologies.	[45]
Water Pollution	Tributyltin	Neurological Disorders	Apoptosis pathways	Endocrine disruptor; induces neuronal apoptosis in vitro.	[77]
Pesticide	Rotenone and Paraquat	Parkinson’s Disease	Mitochondrial Complex I	Inhibit mitochondrial function, causing oxidative stress and dopaminergic neuron death.	[69]

**Table 2:** Major Bioinformatics Tools and Technologies in Environmental Neurogenomics.

Category	Tool/Resource	Description	Application in Environmental Neurogenomics	References
Network Visualization and Analysis	STRING	Protein–protein interactions database integrating experimental and predicted associations.	Helps identify molecular pathways influenced by environmental factors.	[79]
	Cytoscape	Open-source platform for visualizing and analyzing complex biological networks.	Visual analytics for GxE interactions, overlaying multi-omics layers (methylation, expression, protein interactions) in pollution contexts.	[80]
Public Genomic and Epigenomic Databases	ENCODE	Comprehensive annotations of functional genomic elements across diverse tissues and cell types.	Mapping regulatory elements affected by environmental exposure, supporting mechanistic interpretation of epigenetic changes in neural tissues.	[81]
	EWAS Atlas	Curated knowledgebase of epigenome-wide association studies linking CpG sites to phenotypes and exposures	Querying exposure-associated methylation signals, triangulating evidence across cohorts to strengthen causal inference.	[82]
Epigenomic Resources	Gene Expression Omnibus (GEO)	Repository for high-throughput functional genomic data (RNA-seq, DNA methylation and ATAC-seq).	Accessing exposure-response datasets, meta-analyses integrating transcriptomic and epigenomic signatures under pollutant conditions.	[86]
	Roadmap Epigenomics Project	Reference epigenomes encompassing DNA methylation, histone modifications, and chromatin accessibility across human tissues.	Benchmarking exposure related epigenetic alterations, prioritizing candidate loci and pathways relevant to neurodegenerative disease.	[87]

## Data Availability

Data sharing is not applicable to this article as no datasets were generated or analyzed during the current study.
